# TRIM5α SPRY/coiled-coil interactions optimize avid retroviral capsid recognition

**DOI:** 10.1371/journal.ppat.1006686

**Published:** 2017-10-17

**Authors:** Marcin D. Roganowicz, Sevnur Komurlu, Santanu Mukherjee, Jacek Plewka, Steven L. Alam, Katarzyna A. Skorupka, Yueping Wan, Damian Dawidowski, David S. Cafiso, Barbie K. Ganser-Pornillos, Edward M. Campbell, Owen Pornillos

**Affiliations:** 1 Department of Molecular Physiology and Biological Physics, University of Virginia School of Medicine, Charlottesville, Virginia, United States of America; 2 Department of Microbiology and Immunology, Stritch School of Medicine, Loyola University Chicago, Maywood, Illinois, United States of America; 3 Department of Biochemistry, University of Utah, Salt Lake City, Utah, United States of America; 4 Department of Chemistry, University of Virginia, Charlottesville, Virginia, United States of America; Institut Pasteur, FRANCE

## Abstract

Restriction factors are important components of intrinsic cellular defense mechanisms against viral pathogens. TRIM5α is a restriction factor that intercepts the incoming capsid cores of retroviruses such as HIV and provides an effective species-specific barrier to retroviral infection. The TRIM5α SPRY domain directly binds the capsid with only very weak, millimolar-level affinity, and productive capsid recognition therefore requires both TRIM5α dimerization and assembly of the dimers into a multivalent hexagonal lattice to promote avid binding. Here, we explore the important unresolved question of whether the SPRY domains are flexibly linked to the TRIM lattice or more precisely positioned to maximize avidity. Biochemical and biophysical experiments indicate that the linker segment connecting the SPRY domain to the coiled-coil domain adopts an α-helical fold, and that this helical portion mediates interactions between the two domains. Targeted mutations were generated to disrupt the putative packing interface without affecting dimerization or higher-order assembly, and we identified mutant proteins that were nevertheless deficient in capsid binding *in vitro* and restriction activity in cells. Our studies therefore support a model wherein substantial avidity gains during assembly-mediated capsid recognition by TRIM5α come in part from tailored spacing of tethered recognition domains.

## Author summary

TRIM5α is a cytosolic protein that provides effective protection for mammalian cells against retroviral infection. This anti-viral defense mechanism is an unprecedented example of how the cell can recognize entire capsids, which are large, megadalton-sized particles. TRIM5α achieves this by assembling into a hexagonal scaffold that coats the capsid. An important unresolved question is how the capsid-binding SPRY domain of TRIM5α is positioned to optimize its contact points on the capsid surface. Here, we use a variety of techniques to determine that the SPRY domains in the TRIM lattice are organized in pairs and likely to be stably tethered against the hexagonal scaffold. Such an arrangement maximizes the avidity of capsid binding, and allows TRIM5α to act as a “molecular ruler” that matches the spacings and orientations of the capsid subunits.

## Introduction

TRIM5α is a restriction factor that recognizes and binds the incoming cores of retroviruses such as HIV [1–3], and represents a first-line intracellular antiviral defense mechanism. Upon core binding, TRIM5α induces accelerated capsid dissociation or uncoating, inhibits reverse transcription, and activates innate immune signaling pathways [1, 3, 4]. Like other members of the TRIM family [5], TRIM5α contains a tripartite or RBCC motif at its N-terminus (RING, B-box 2, and coiled-coil domains)–the RING domain mediates E3 ubiquitin ligase effector functions required to inhibit reverse transcription and signal interferon [4, 6, 7], whereas the coiled-coil and B-box 2 domains respectively mediate TRIM5α dimerization and higher-order assembly [8–16]. The TRIM5α RBCC motif is connected by a long linker (L2 or linker 2) to a C-terminal SPRY domain that directly contacts retroviral capsids [1–3, 17].

Retroviral capsids are higher-order macromolecular assemblages composed of about 1,500 viral CA protein subunits, which assemble on a hexagonal lattice of several hundred hexamers and 12 pentamers [18, 19]. Accordingly, TRIM5α also undergoes higher-order assembly in order to bind retroviral capsids [10, 20]. Although an individual SPRY domain does not have appreciable affinity for the capsid (estimated to be in the mM range [21]), dimerization of the coiled-coil domain [8, 9, 12] and trimerization of the B-box 2 domain [15, 16, 20, 22] creates a hexagonal TRIM lattice that displays an array of SPRY domains for multivalent, avid capsid binding [10, 23]. This “pattern recognition” model and the architecture of the TRIM lattice are supported by structural and biochemical studies of *in vitro* TRIM5α/capsid complexes and crystal structures of individual domains and fragments of TRIM5α and other TRIM proteins [3, 8–16, 20, 22, 23]. However, the molecular details of SPRY domain positioning–whether it is flexibly displayed or tethered–remain experimentally undefined. This unresolved issue is a core concept of the avidity-driven recognition mechanism.

The L2 linker that connects the SPRY domain to the coiled-coil is likely to facilitate positioning of the SPRY domain. Mutagenesis studies have shown that an intact L2 sequence is required for efficient retroviral restriction [24, 25], and L2 polymorphisms have been reported to correlate with susceptibility to HIV-1 infection [26]. Furthermore, evolutionary sequence analysis has shown that some L2 residues are under positive selection, even though the linker itself does not contact the capsid [17]. In crystal structures of TRIM protein dimerization domains, the L2 linkers display substantial degrees of disorder but have been seen to fold into a short C-terminal helix that packs against the center of the coiled-coil dimer [11–14]. This packing interaction can be therefore quite flexible, and this flexibility has been proposed to underlie degenerate binding of the SPRY domain to capsid surface epitopes as well as a mechanism to destabilize the capsid lattice [11–13, 27, 28]. Alternatively, it has been proposed that the C-terminal L2 helix is integrated with the downstream SPRY domain fold, and that this L2/SPRY helix packs more stably against the coiled-coil helices and thereby positions two SPRY domains at a defined spacing and orientation relative to each other [11, 12]. This issue has not yet been resolved, in part because structures of TRIM5α constructs containing the coiled-coil, L2, and SPRY domains have been notoriously difficult to obtain. Here, we describe biochemical, biophysical, and cell biological experiments to test the models for SPRY domain positioning. Our results are consistent with a tethered mechanism: the residues at the L2/SPRY boundary are indeed helical, and packing of this helix to the main coiled-coil helix not only facilitates capsid recognition, but also modulates stability of the TRIM5α dimer, efficiency of higher-order assembly, and overall antiviral activity.

## Results

### Packing of the L2/SPRY helix against the coiled-coil helix

**Fig 1:**
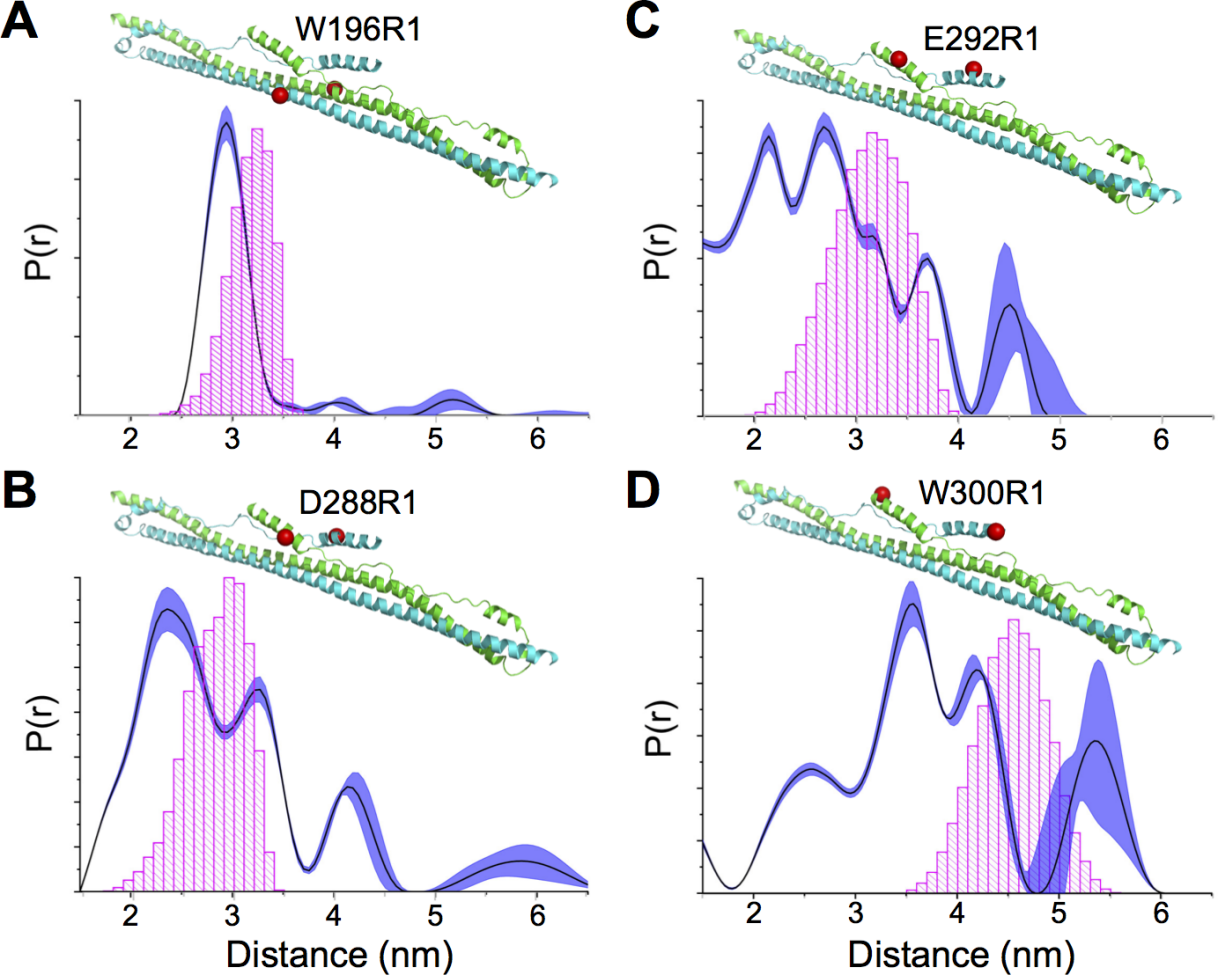
Conformationally heterogeneous packing of the terminal L2 helix against the coiled-coil scaffold in context of the CC-L2 fragment of rhesus TRIM5α. (A-D) Distance distribution curves show a single well-defined peak when R1 is attached to coiled-coil helix (W196R1). Multiple peaks are obtained when labels are appended to the indicated L2 helix residues (D288R1, B; E292R1, C; W300R1, D). Note that since the protein is a dimer, a single cysteine substitution can be used to attach two labels for distance measurements. The shaded regions represent fits to the DEER data that are within 15% root mean square deviation of the best fit (dark trace). Corresponding time-traces and subtracted dipolar evolutions are shown in S1 Fig. Expected distances calculated from the static structural model are indicated by the histograms in magenta. These are based upon all the available rotamers given the steric constraints in the model.

In the published crystal structure of the TRIM5 B-box 2/coiled-coil/L2 fragment, both of the subunits in the antiparallel dimer had substantial disorder in their L2 regions, but in one subunit the L2 C-terminus was folded into a short α-helix [12]. Crystal structures of other TRIM proteins displayed similar variations in L2 configurations, and some of the variations appeared to have been caused by crystal packing interactions [11, 13]. We therefore first sought confirmation that L2 packed against the coiled-coil helix in solution, using site-directed spin labeling and paramagnetic double electron-electron resonance (DEER) spectroscopy (Fig 1 and S1 Fig). In this experiment, the distance of separation between a pair of labels can be determined provided that phase modulation can be reliably measured (<8 nm) [29]. Measurements were performed on a purified recombinant CC-L2 fragment of rhesus TRIM5α (residues 133–300) that includes the full sequence of the putative L2/SPRY helix (_281_PDLKGMLDMFRELTDARRYW_300_) [11].

In a control experiment, we first confirmed that labels appended to the main coiled-coil helix (W196R1) had a single distance distribution peaking at the expected distance–about 3 nm–between the two labels (Fig 1A). In contrast, labels appended to the terminal L2 helix (D288R1, E292R1, W300R1) returned progressively broader distance distributions with multiple peaks as the labels approached the C-terminus (Fig 1B–1D). These are indicative of either a dynamic helical configuration or dynamic packing of the L2/SPRY helix against the coiled-coil. These results are consistent with the crystal structures [11–13], as well as a recent biochemical analysis of the CC-L2 fragment of rhesus TRIM5α [28].

**Fig 2:**
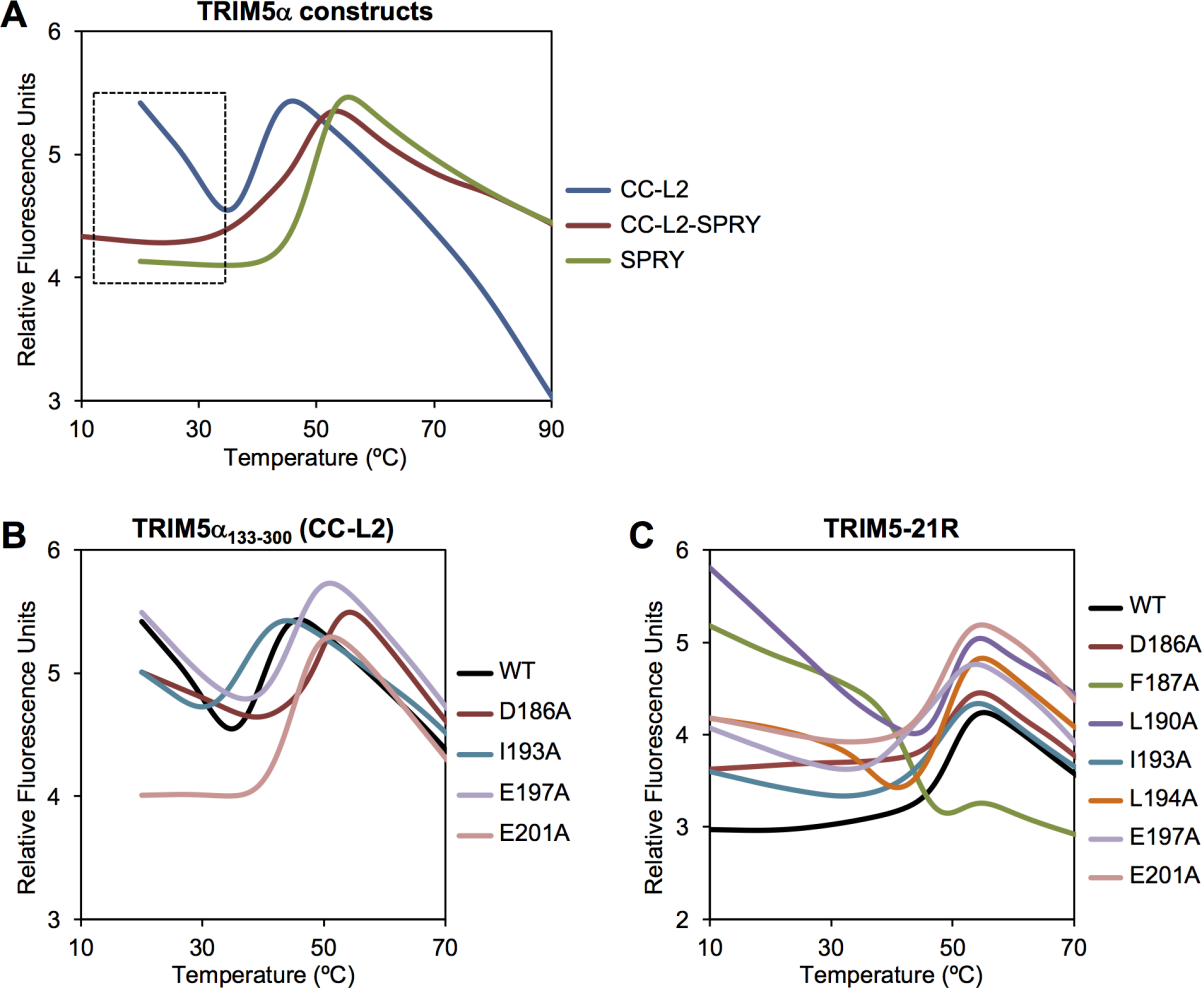
Differential scanning fluorimetry thermal stability profiles of purified TRIM5α proteins. (A) Comparison of CC-L2, CC-L2-SPRY, and SPRY constructs. (B) Effect of model-based mutations in context CC-L2. (C) Effect of model-based mutations in context of the restriction-competent TRIM5-21R protein.

Given the likelihood that L2 disorder in CC-L2 was caused by the absence of the SPRY domain, it was important to perform a comparative DEER analysis with a protein construct containing an intact SPRY. Unfortunately, we were unable to perform these experiments because the recombinant CC-L2-SPRY protein did not tolerate removal of its 7 native cysteine residues to allow for site-directed thiol-based labeling. We therefore determined the effect of the SPRY domain on L2 flexibility by comparing CC-L2 and CC-L2-SPRY stabilities using a thermal melting experiment called differential scanning fluorimetry. In this assay, protein unfolding is monitored with a dye that fluoresces upon binding hydrophobic residues that become exposed with increasing temperature [11]. As shown in Fig 2A, the CC-L2 construct (blue curve) displayed the expected melting profile for a coiled-coil protein, with a single transition reflecting the coupled folding and dimerization; the apparent melting point (*T*_m_) was about 41°C. The SPRY domain alone also displayed a single transition, consistent with its monomeric configuration in isolation, with a *T*_m_ of about 50°C (green curve). The CC-L2-SPRY protein (maroon curve) had an intermediate *T*_m_ about halfway between CC-L2 and SPRY. Most notably, the CC-L2 profile had significantly elevated signals at the start of the experiment, which is distinct from the flat profiles of CC-L2-SPRY and SPRY (boxed area in Fig 2A). The elevated signals indicated that CC-L2 had exposed hydrophobic residues even at low temperature. The simplest interpretation of this observation is that the L2 linker was undergoing dynamic packing (association and dissociation) with the coiled-coil in this construct. Conversely, the flat signals for CC-L2-SPRY suggested that in the presence of the SPRY domain, L2 is more stably packed against the coiled-coil.

### N-terminal extensions of the SPRY domain have helical propensity

**Fig 3:**
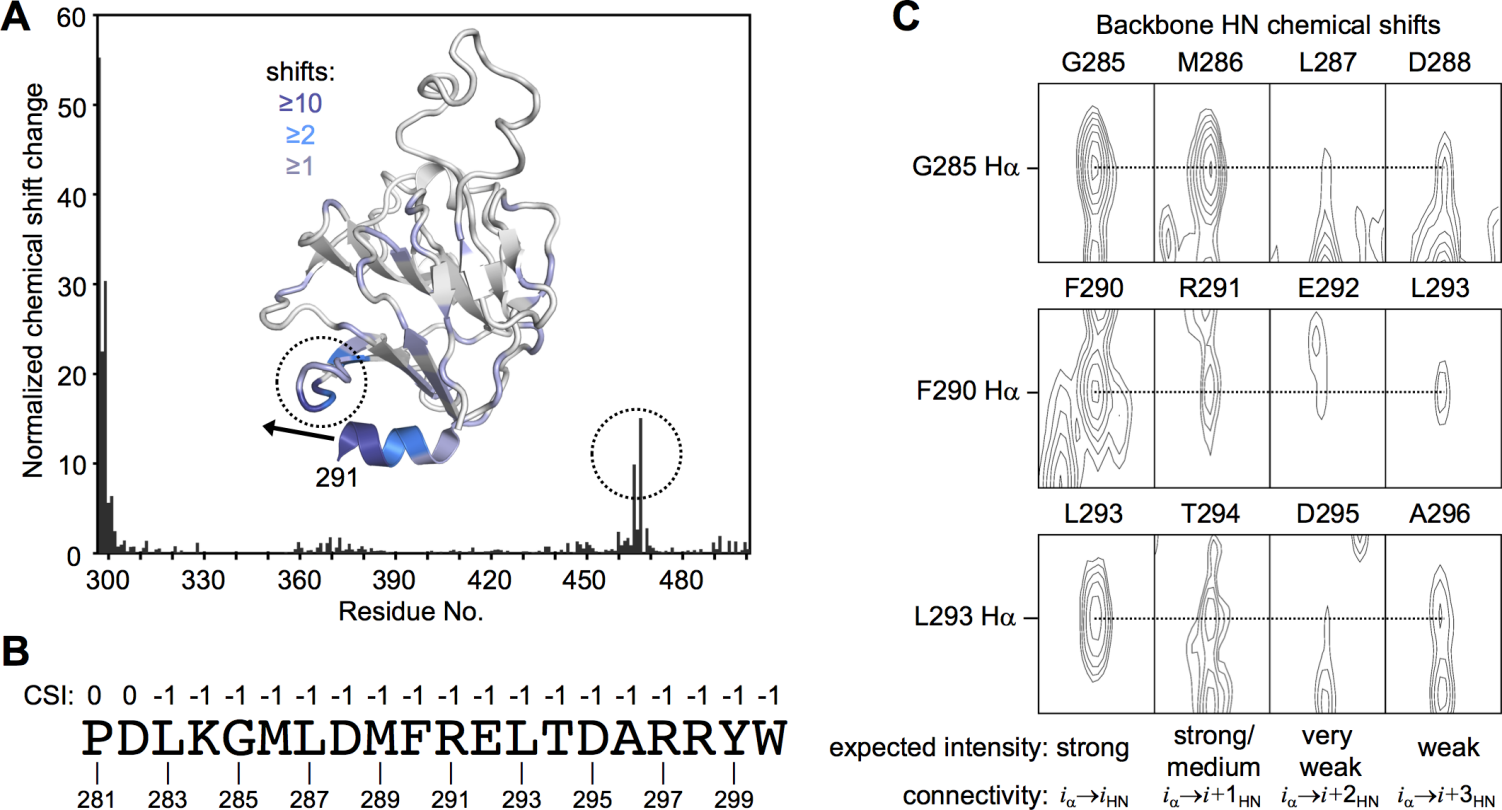
NMR analysis of N-terminal extensions of the isolated TRIM5α SPRY domain. (A) Normalized backbone amide chemical shift differences in comparing rhesus TRIM5α constructs spanning residues 292–497 and 281–497. Chemical shift changes were also mapped onto the SPRY domain structure (PDB 2LM3 [30]). Dashed circles highlight the loop that undergoes the greatest changes, apart from the N-terminal residues. Black arrow indicates the trajectory of the putative extended helix. (B) Normalized chemical shift indices calculated from assigned Cα, Cβ, C (carbonyl), and Hα resonances by using the program PREDITOR [64], shown for each of the indicated residues in the putative L2/SPRY helix. Values indicate predicted secondary structure: 1 = β-strand, 0 = random coil, -1 = α-helix. (C) Sections of an ^15^N-edited 3-dimensional NOESY spectrum with well-resolved sequential Hα-HN cross-peaks. Resonance overlap precluded identification of cross-peaks for the entire helical sequence.

We next performed a complementary analysis of the putative L2/SPRY helix in context of the isolated SPRY domain. Published structures of the TRIM5α SPRY domain have revealed that residues 292–300 (the C-terminal half of the L2/SPRY boundary) are indeed helical and appear to pack stably against the main body of the domain [30, 31]. However, in one of the structures, residues that form part of the predicted helix (287–291) adopt a non-helical, random coil configuration [31]. To test whether these and additional N-terminal residues would actually adopt a helical configuration in solution, we used NMR spectroscopy to analyze a SPRY construct starting at residue 281 and compared this to a truncated construct starting at residue 292. Control spectra indicated that the two SPRY proteins had the same fold (S2 Fig). Importantly, we found three complementary indications that the additional residues in the longer construct were likely to be helical. First, significant chemical shift perturbations were observed for residues located in a loop (encircled in Fig 3A) that would physically encounter the extended helix. Second, analysis of chemical shift deviations from random coil values for backbone carbon and proton atoms indicated the presence of contiguous α-helical secondary structure in the segment spanning residues 283–300 (Fig 3B). Third, we observed three 4-residue segments (_285_GMLD_288_, _290_FREL_293_, and _293_LTDA_296_) with fortuitously well-resolved sequential proton-amide cross-peaks in an ^15^N-filtered NOESY spectrum (Fig 3C). The absolute peak intensities obey the expected sequential pattern for α-helical segments, with strong/medium *i*→*i*+1 cross-peaks, very weak *i*→*i*+2, and weak *i*→*i*+3. Collectively, these data indicated that the helical termini observed separately in the crystal structures of the CC-L2 and SPRY fragments of TRIM5α probably constitute a single, contiguous helix in the full-length protein.

### Modeling of the putative coiled-coil/L2/SPRY packing interface

**Fig 4:**
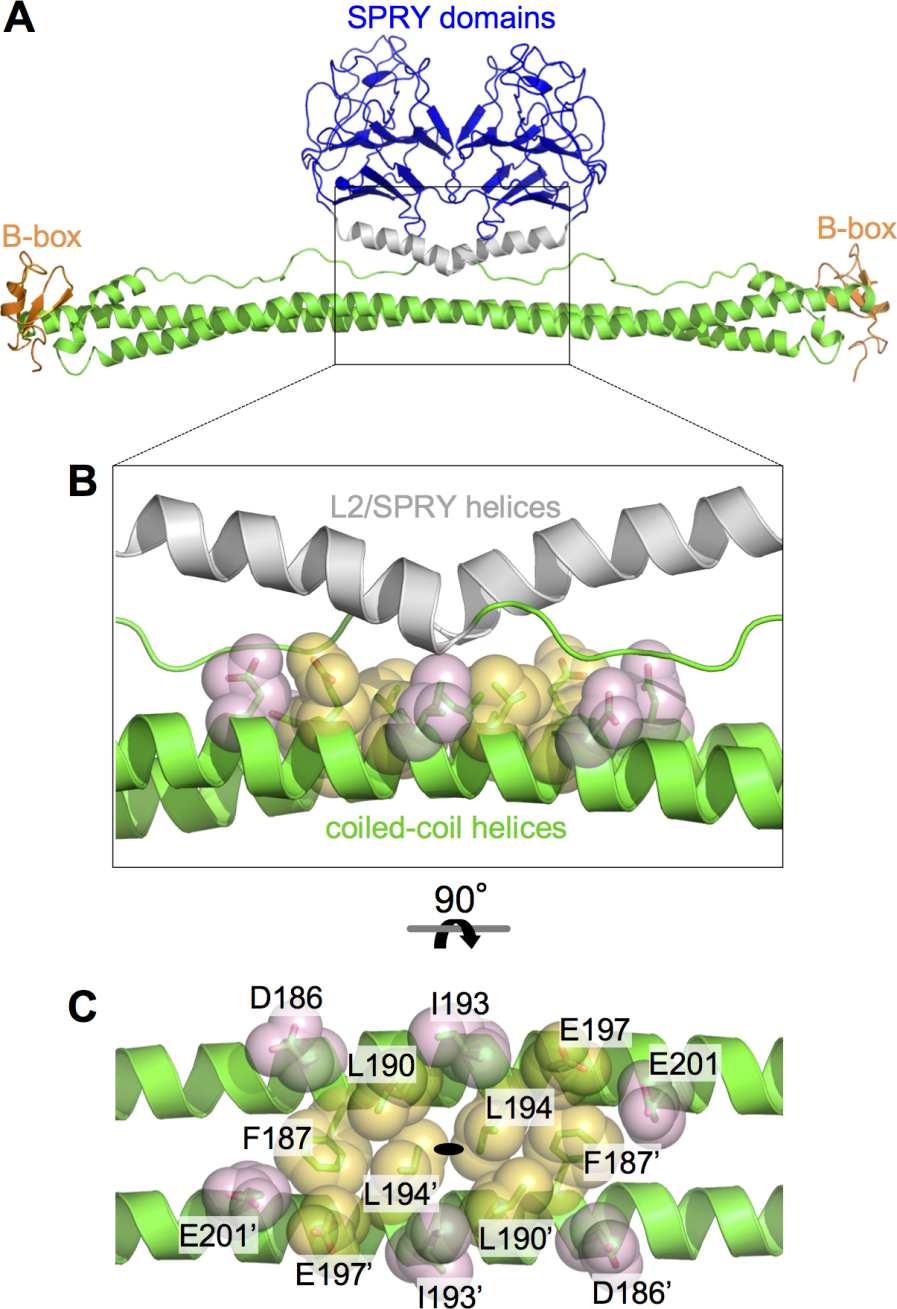
Modeling of SPRY/coiled-coil packing in TRIM5α. (A) Ribbon model of the TRIM5α dimer, which was obtained by combining the crystal structures of the B-box/coiled-coil dimer [12] and isolated SPRY domain [30, 31]. Domains and structural elements are colored as follows: RING, not included in model; B-box 2, orange; coiled-coil, green; L2 linker helix, gray; SPRY, blue. (B) Expanded view of the central region of the antiparallel dimer, in the same orientation as A. Residues selected for mutagenesis in this study are represented by sticks and transluscent spheres. Orange, class I residues; pink, class II residues. (C) Orthogonal view rotated as indicated, with individual residues labeled. The L2 helices are omitted in this panel for clarity. The two subunits are distinguished by an apostrophe. The coiled-coil dimer’s two-fold symmetry axis is indicated by the black oval.

Given the above results and in the absence of an experimentally determined structure as yet, we computed a molecular model of the CC-L2-SPRY dimer (Fig 4A) and designed a mutagenesis study to test it. The model was built by first symmetrizing the B-box 2/coiled-coil/L2/lysozyme structure [12] to obtain ordered L2 regions for both subunits in the dimer, and then modeling a contiguous helix spanning residues 283–300 in the L2/SPRY boundary by superimposing matching residues in the isolated SPRY domain structure [30]. In this model, the orientations of the SPRY domains were dictated primarily by interactions between the two L2/SPRY helices and the middle of the two coiled-coil helices, as previously suggested [11, 12]. Given significant model uncertainties in the positions of the L2/SPRY residues in the interface, we focused on the reliably defined coiled-coil residues for mutagenesis. Fourteen coiled-coil residues (7 per subunit) were buried within the putative packing interface, and we selected these for alanine substitution (Fig 4B and 4C). For biochemical experiments, mutations were made in the CC-L2 fragment described above and in TRIM5-21R, a chimeric construct described in previous studies as a useful recombinant surrogate for TRIM5α [8–10, 15, 23, 32]. Mutations were also made in context of full-length rhesus TRIM5α in a mammalian expression vector for analysis of assembly and restriction phenotypes in cells. The collective functional data are summarized in Table 1.

**Table 1:** Functional phenotypes of TRIM5α mutants.

Mutation	Class	Tube Binding	*In Vitro* Assembly	Cytoplasmic Bodies	Restriction Activity
None (WT)		++	++	++	++
D186A	II	+	n.d.*	+	+
F187A	I	–	n.d.	–	–
L190A	I	+	n.d.	+/–	–
I193A	II	–	++	++	+
L194A	I	+	n.d.	–	–
E197A	I	+	n.d.	+/–	+
E201A	II	–	++	++	+

* n.d. = not determined

### Effects of mutations on protein dimerization and stability

Studies of the related protein, TRIM25, have shown that single alanine substitutions in the center of the elongated coiled-coil helix can severely destabilize the dimer, even though the dimerization interface is quite extensive [11]. We therefore used the thermal melting assay above to determine which of the model-based TRIM5α mutants were deficient in dimerization. In context of the CC-L2 construct, we found that the F187A, L190A, and L194A mutants were very prone to aggregation and could not be purified easily, indicating that the mutations severely destabilized the dimer. These results are consistent with their positions within the interface, as these three hydrophobic residues bridge contacts between the coiled-coil helices as well as between the coiled-coil and L2/SPRY helices (Fig 4B and 4C). Similar results were observed for equivalent mutations in the CC-L2 dimer of TRIM25 [11]. In contrast, the remaining mutations produced purifiable CC-L2 proteins. Of these, I193A measurably reduced the stability of the dimer (*T*_m_ = 37°C), whereas the others (D186A, E197A, E201A) had no effect or even slightly increased the apparent *T*_m_ compared to the wildtype control (Fig 2B).

**Fig 5:**
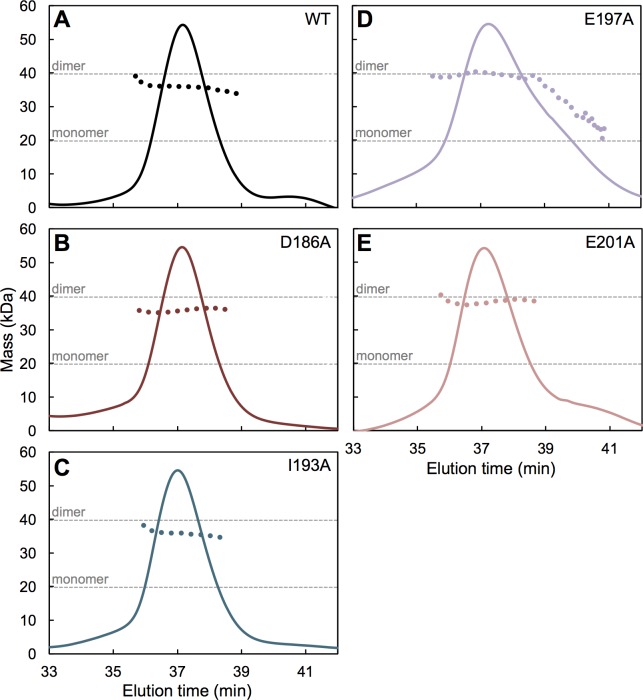
Dimerization of CC-L2 mutants. Purified mutant proteins were analyzed by using SEC-MALS (size exclusion chromatography coupled with multi-angle light scattering). The solid curves represent the normalized UV absorbance trace (arbitrary units) of eluting components. The dotted curves show the population averaged molecular mass calculated from the measured protein concentration and light scattering data. Dashed gray lines indicate the expected masses of the monomer and dimer species. (A) Wildtype control. (B) D186A. (C) I193A. (D) E197A. The major peak had a substantial trailing edge indicating dissociation into monomers. (E) E201A.

To more rigorously determine oligomerization states, we also analyzed the CC-L2 mutants by using SEC-MALS (size exclusion chromatography coupled with multi-angle light scattering). Consistent with the thermal shift data, SEC-MALS showed that D186A (Fig 5B), I193A (Fig 5C), and E201A (Fig 5E) were dimeric just like wildtype control (Fig 5A). The E197A mutant was likewise dimeric, but the major peak also had a significant trailing shoulder indicating dissociation into monomer (Fig 5D). Thus, this CC-L2 mutant was also deficient in dimerization, although not to the same extent as the F187A, L190A, and L194A mutants.

We next tested the mutations in context of TRIM5-21R, a restriction-competent chimeric construct wherein the RING domain of rhesus TRIM5α has been replaced by that of human TRIM21 [32, 33]. It was previously shown that this protein expresses both as a monomer and dimer–the two oligomers can be cleanly separated by sequential anion exchange and size exclusion chromatography steps (S3A and S3B Fig) [8–10]. In this case, all the mutants were purifiable, but the monomer fractions of F187A, L190A, L194A, and E197A during initial purification steps comprised 50% or more of the total protein, consistent with significant defects in dimerization (S3C Fig). In contrast, the D186A, I193A, and E201A mutants were more similar to wildtype, with the dimers being the major fraction (S3D Fig). For thermal melting analysis, we purified the dimer fraction for each mutant. Compared to the CC-L2-SPRY construct, the melting curve of wildtype TRIM5-21R had a sharper transition and higher *T*_m_ of 50°C (Fig 2C, black curve). The increased stability is likely due to “capping” of the coiled-coil ends by the B-box 2 domains, as observed in crystal structures [12, 15, 16]. Consistent with severe destabilization of the F187A, L190A, and L194A CC-L2 constructs, the equivalent TRIM5-21R proteins were still clearly unstable, with high signals at early time points similar to the wildtype CC-L2 construct (Fig 2C). In particular, F187A, which eliminated a significant proportion of the hydrophobic core, produced a non-canonical melting profile (Fig 2C, green curve). The remaining mutants did not significantly perturb the *T*_m_ of the TRIM5-21R dimer, but also had elevated signals at low temperature, which we interpret to mean that the mutations also weakened packing of L2/SPRY against the coiled-coil, as predicted by the computational model.

On the basis of the thermal melting and chromatography data, we classified mutations within the putative CC/L2/SPRY interface into two groups: class I mutants (F187A, L190A, L194A, and E197A; orange in Fig 4B and 4C) had significant or measurable effects on dimerization, whereas class II mutants (D186A, I193A, and E201A; pink in Fig 4B and 4C) had little or no effect on dimerization but still likely important for CC/L2/SPRY packing. We therefore considered this second group to be more informative in the experiments below.

### Effects of mutations on capsid binding activity *in vitro*

**Fig 6:**
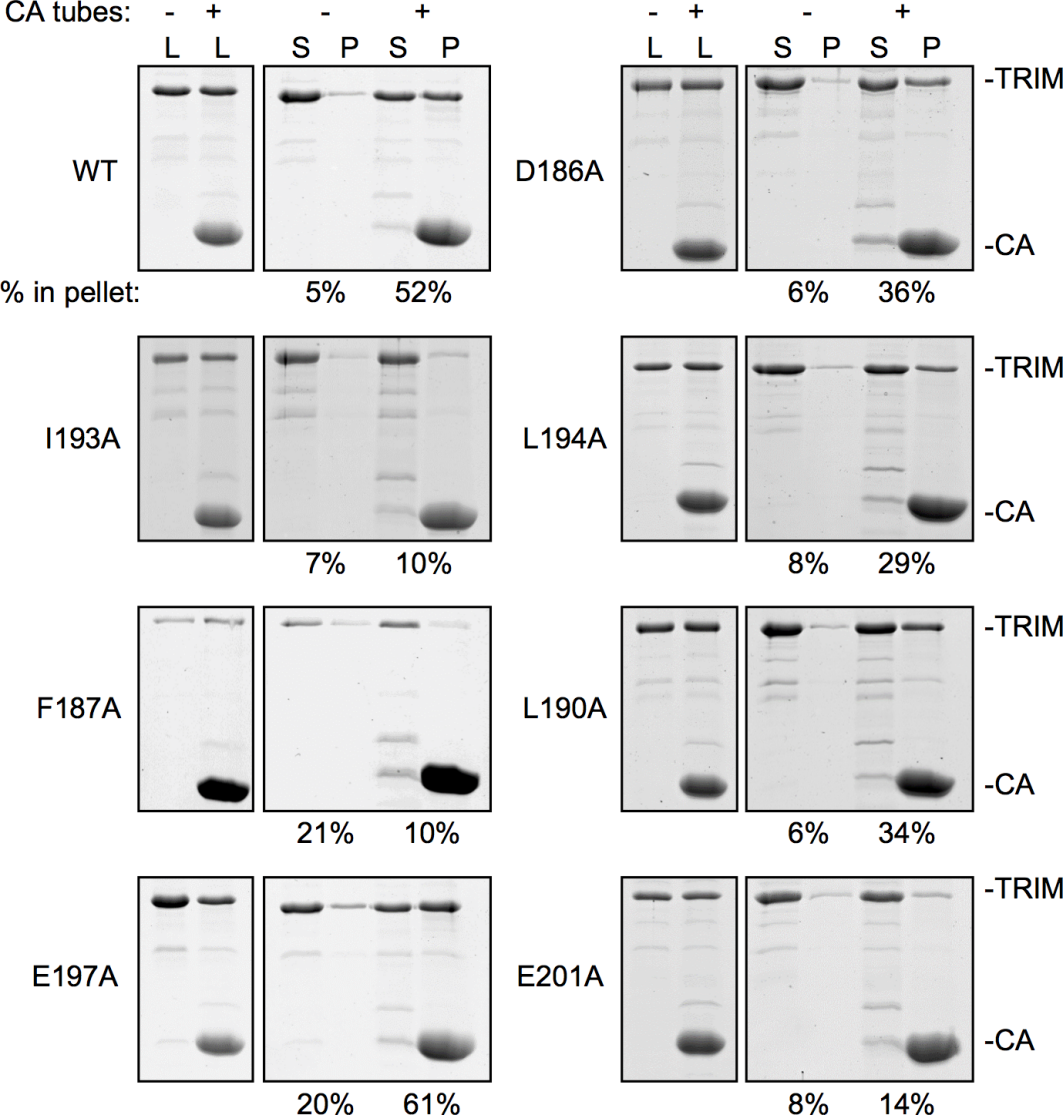
Capsid binding activities of TRIM5-21R proteins. Representative results of pull-down assays. Purified TRIM5-21R (5 μM) was incubated with disulfide-stabilized HIV-1 CA tubes, fractionated by centrifugation, and visualized by SDS-PAGE with Coommassie staining. L, load; S, soluble fraction; P, pellet fraction. Band intensities were quantified by densitometry. Experiments were repeated at least 2 times for each mutant using independent protein preparations, with similar results.

If the positions of the SPRY domains in the TRIM5α dimer were tailored to match the spacing of epitopes on retroviral capsids, then destabilization of coiled-coil/L2/SPRY packing would also disrupt capsid recognition. We therefore tested the mutant TRIM5-21R proteins for their ability to bind disulfide-stabilized HIV-1 CA tubes by using an established centrifugation assay [3, 10, 15, 34]. Using our specific protocol, about 50% of wildtype TRIM5-21R was consistently found to co-pellet with the CA tubes [15]. Given the propensity of TRIM5-21R to spontaneously assemble and the reduced stabilities of the mutants, experiments were performed right after purification. Each mutant was analyzed at least twice with independent protein preparations, and always in parallel with a wildtype control. Representative results are shown in Fig 6.

Consistent with expectation that the dimer is the minimal capsid-binding unit of TRIM5α, the F187A class I mutant pelleted only at background levels. The L190A and L194A mutations had less severe defects (with 34% and 29% pellet, respectively), consistent with the less severe biophysical defects observed in the thermal stability assays. E197A pelleted efficiently with the tubes (61%), although we consider this mutant to be also an intermediate binder because in four independent experiments it reproducibly showed high levels of background pelleting (20–30%), likely due to aggregation. For reference, these levels of residual binding are similar to those observed for the R121E and W117E mutations, which disrupt higher-order interactions mediated by the B-box 2 domain and essentially abolish restriction activity [10, 15]. Among the class II mutants, D186A had significant residual binding (36%), whereas I193A and E201A only pelleted at background levels. Thus, there is good correlation between the expected structural effects of the mutations, the biophysical properties of the mutant proteins, and their capsid binding activities.

### Effects of mutations on higher-order assembly

**Fig 7:**
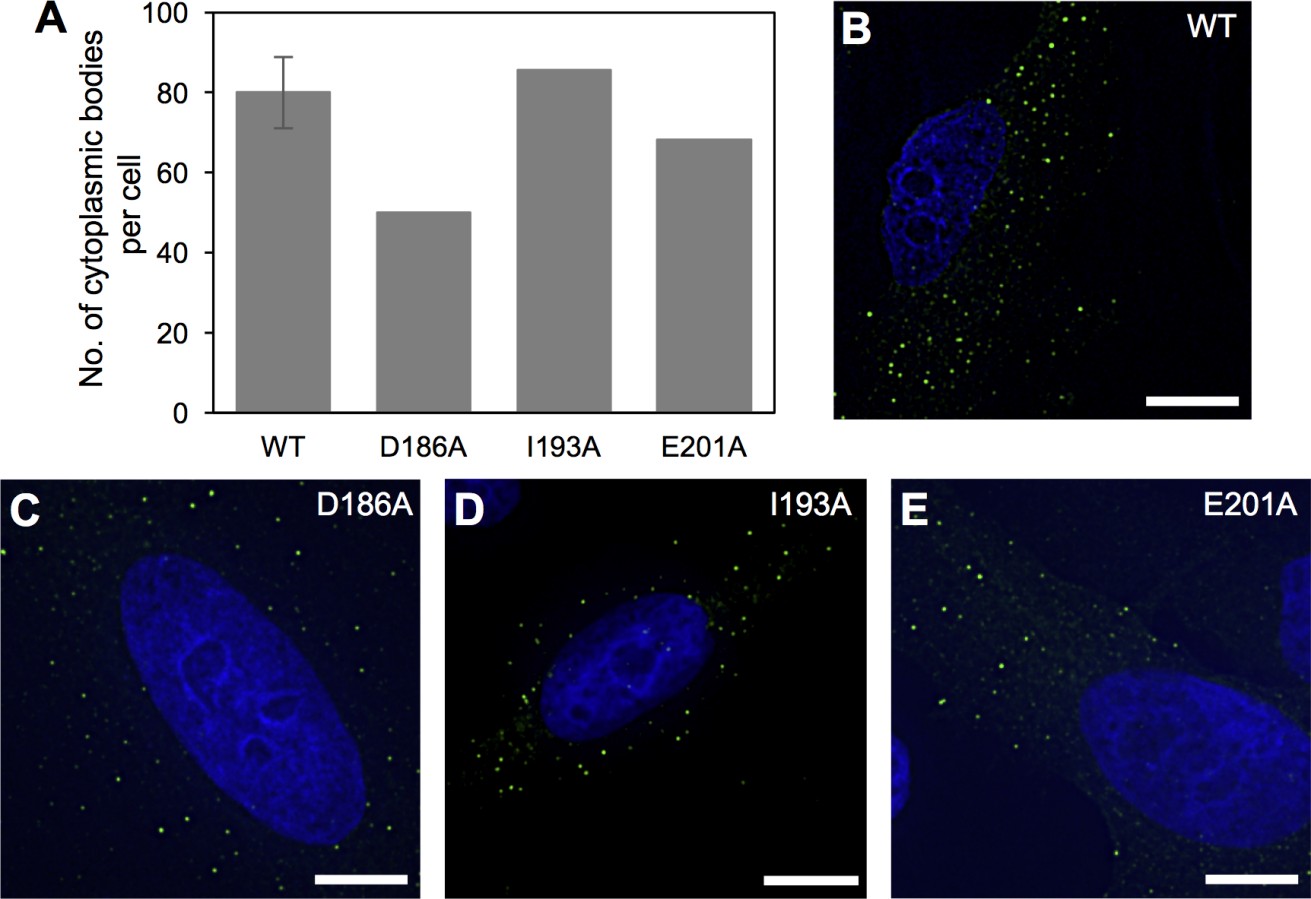
Cytoplasmic body assembly activities of YFP-TRIM5α proteins. (A) The number of cytoplasmic bodies was counted in each cell and normalized to the intracellular YFP concentration as described [24]. (B) Representative image of HeLa cells stably expressing the wildtype control. (C) D186A. (D) I193A. (E) E201A. Cytoplasmic bodies appear as green puncta. DAPI was used to stain nuclei blue. Scale bars = 10 μ.

Mutations in the L2 linker have been previously shown to disrupt TRIM5α self-association and higher-order assembly [24, 25, 27, 35]. To test the alternative possibility that the capsid binding defects we observed simply reflected this property, we overexpressed YFP-tagged rhesus TRIM5α in HeLa cells and tested the mutants for their ability to form fluorescent puncta called cytoplasmic bodies. Although not yet definitively proven, these cytoplasmic bodies are reasonably believed to reflect the intrinsic ability of purified TRIM5α proteins to assemble *in vitro* [1, 10, 15]. In these experiments, the wildtype control produced around 80–100 individual puncta per cell (Fig 7A and 7B). As expected, the class I mutants that were appreciably deficient in dimerization were also significantly impaired in cytoplasmic body formation (S4 Fig). F187A and L194A, which were the most severe mutations in our *in vitro* assays, produced virtually no cytoplasmic bodies (S4A and S4C Fig), again confirming that the dimer is the fundamental building block of higher-order assemblies of TRIM5α.

**Fig 8:**
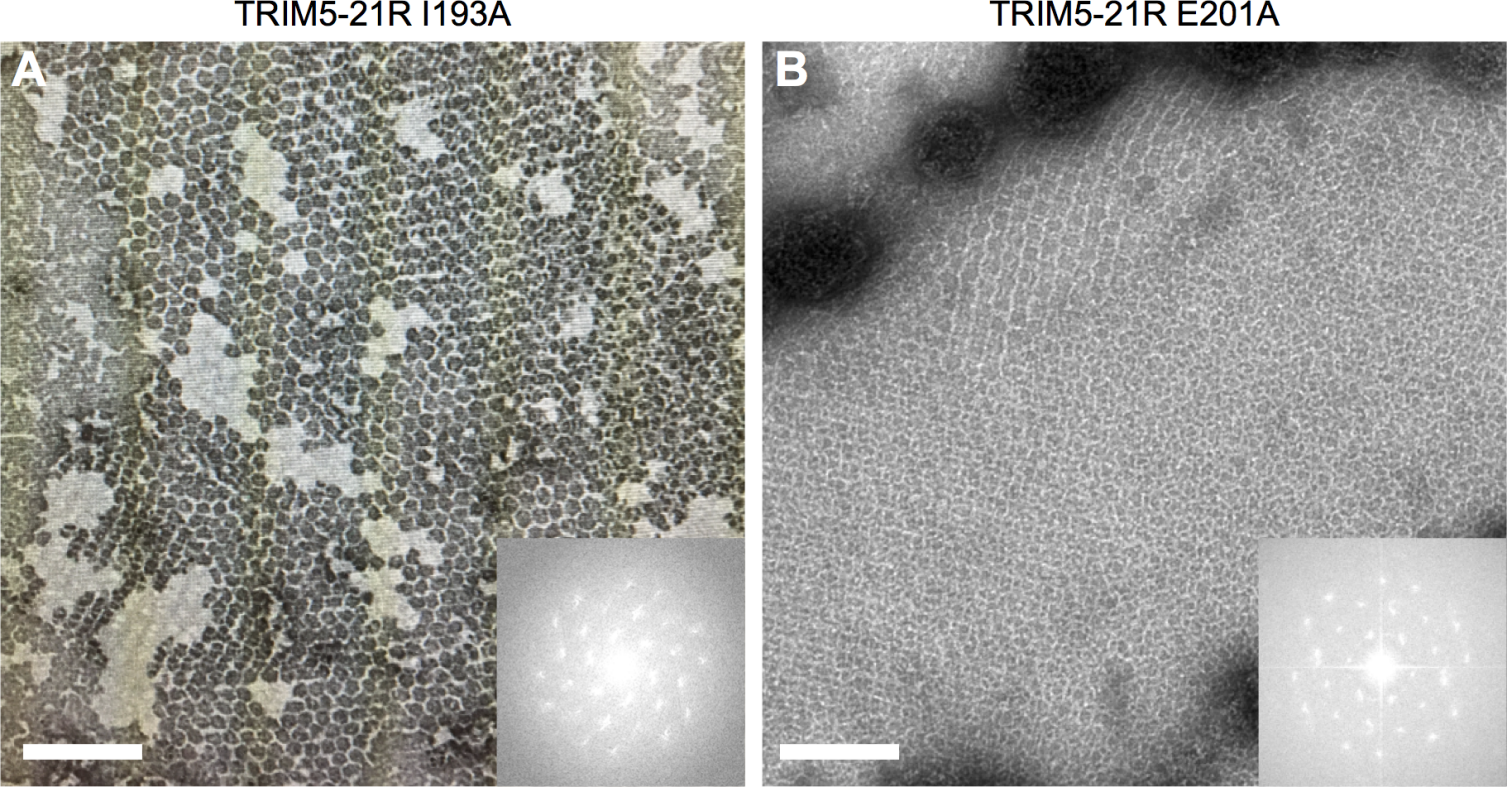
*In vitro* assembly activities of class II TRIM5-21R mutants. Purified TRIM5-21R proteins were incubated in assembly buffer overnight and the resulting precipitates were examined by negative stain electron microscopy. (A) I193A. (B) E201A. Insets: Fourier transforms of the associated images. Scale bars = 200 nm.

Results also showed that the three class II mutants retained the ability to assemble into cytoplasmic bodies (Fig 7). Importantly, I193A and E201A, which showed only background levels of capsid binding *in vitro*, assembled puncta about as efficiently as wildtype (Fig 7D and 7E). To correlate these results with *in vitro* assembly phenotypes, we assembled purified TRIM5-21R harboring these two mutations, and confirmed that both mutant proteins efficiently assembled into a large hexagonal lattice with the expected unit cell dimensions (Fig 8) [10, 15, 23]. These results also provide further evidence that the mutants were stably dimeric, because monomeric TRIM5-21R is severely impaired in assembly *in vitro* [10]. We therefore conclude that the significant binding defects caused by the I193A and E201A mutations were not due to disruption of dimerization or higher-order assembly, but more likely due to impaired positioning of the SPRY domain relative to the coiled-coil domain.

### Effects of mutations on restriction activity

**Fig 9:**
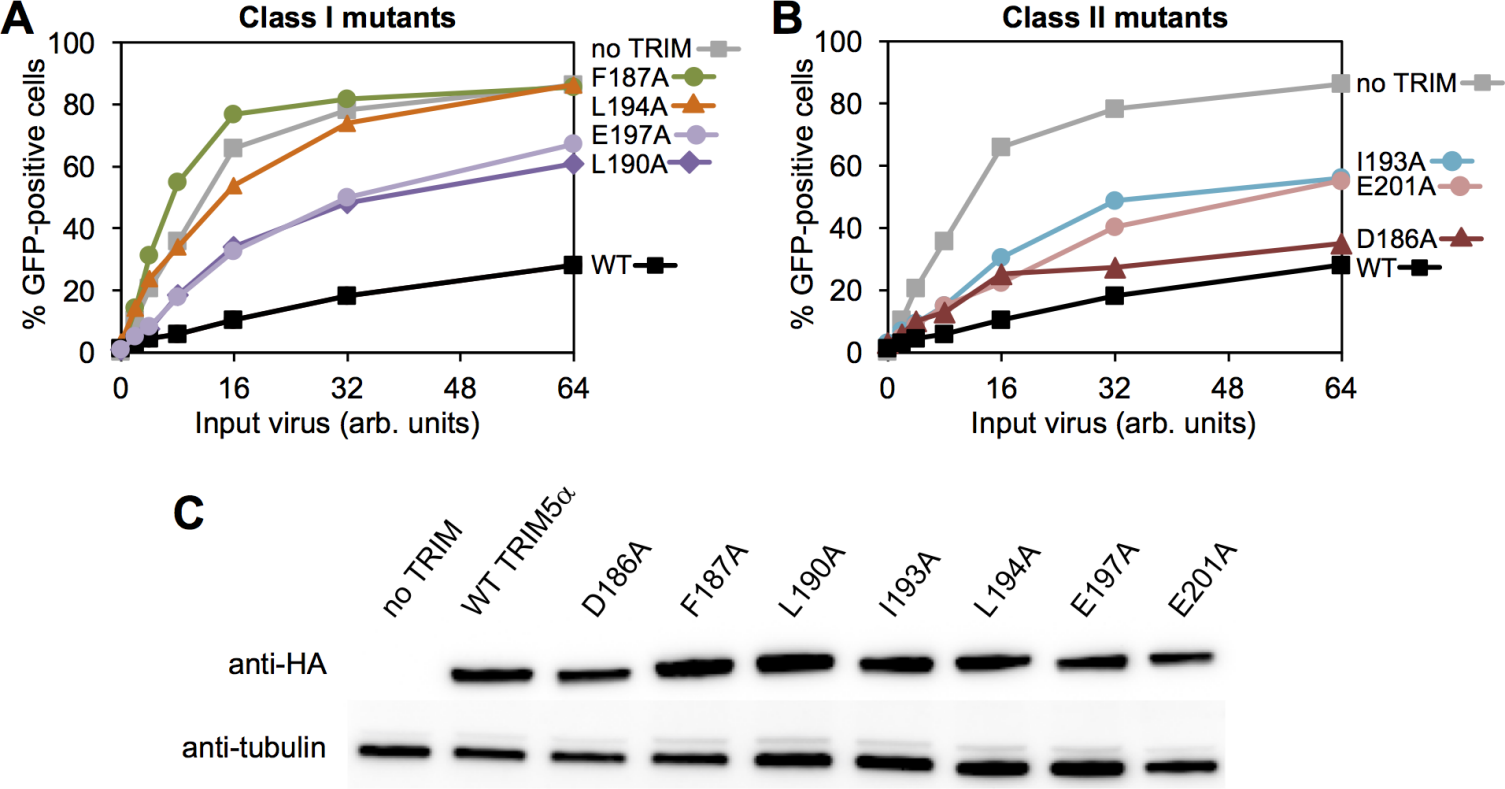
Restriction activities rhesus TRIM5α proteins. HeLa cells that stably expressed the indicated HA-tagged TRIM5α proteins were infected with GFP-labeled HIV and the extent of viral replication was quantified. (A) Class I mutants. (B) Class II mutants. (C) Expression levels were quantified by immunoblotting. Experiments were repeated 6 times independently with similar results.

We then determined the ability of our mutants to inhibit HIV-1 replication in cultured HeLa cells. Consistent with expectation from the above analysis, the class I mutants were significantly impaired in restriction, and the extent of impairment correlated with the degree to which each mutant was deficient in dimerization *in vitro* (Fig 9A). Of the class II mutations, D186A only had a minor defect in restriction (Fig 9B, maroon), which correlated with its *in vitro* properties and intermediate defect in the capsid-binding assay (Fig 6). This residue is located at the outer edges of the modeled SPRY/coiled-coil interface, and therefore probably does not significantly contribute to the packing interaction (Fig 4C). In contrast, the I193A and E201A mutants were more significantly impaired in restriction (Fig 9B, blue and pink), which again correlated with more severe loss of binding activity *in vitro* and more significant interactions with the L2/SPRY helix in the modeled TRIM5α dimer (Fig 4B and 4C). Note, however, that both the I193A and E201A mutants still retained measurable levels of antiviral activity. We speculate that the relatively high protein expression levels in our stably transfected cell lines, combined with the intact ability of the mutant proteins to form higher-order assemblies, may have buffered the effects of the mutations (see also Discussion below).

**Fig 10:**
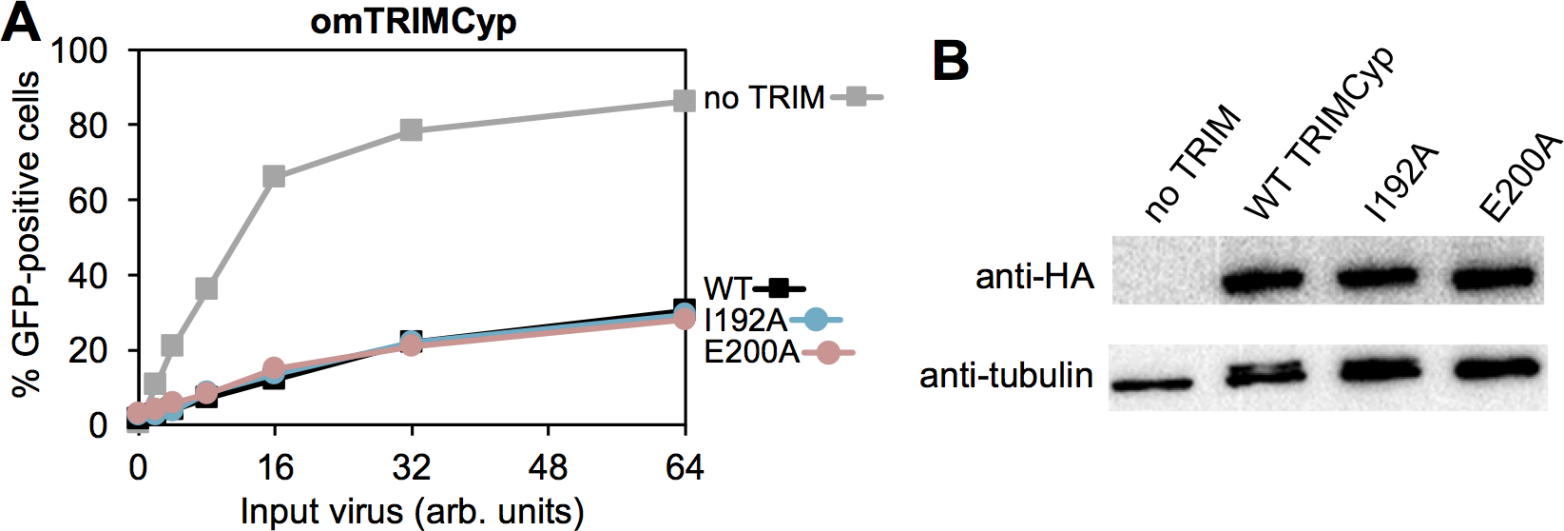
Restriction activities of TRIMCyp proteins. (A) HeLa cells that stably expressed the indicated HA-tagged owl monkey TRIM5α proteins were infected with GFP-labeled HIV and the extent of viral replication was quantified. (B) Expression levels were quantified by immunoblotting. Experiments were repeated 2 times independently with similar results.

Finally, we tested the mutations in context of owl monkey TRIMCyp, which we predicted would be relatively insensitive to positioning effects due to its higher intrinsic affinity for the HIV-1 capsid protein [36–40]. Indeed, both the TRIMCyp I192A and E200A mutants (equivalent to TRIM5α I193A and E201A) were just as restriction-competent as the wildtype control (Fig 10). Importantly, these results also help exclude pleiotropic effects of the mutations.

## Discussion

Multivalency is commonly found in nature to achieve tight binding, even though each component univalent interaction is by itself very weak, by bonding or linking together multiple copies of the interacting subunits. In these systems, simple clustering of binding domains can already result in significant binding, but truly substantial avidity gains are observed when the spacing of tethered recognition domains is matched to the spacing of their corresponding epitopes [41]. Here, we provide experimental evidence that in the TRIM5α dimer, the two SPRY domains are tethered by an α-helical segment that integrates into the SPRY domain fold and packs against the center of the coiled-coil scaffold. The simplest interpretation of our structural and biophysical data (and those of others) is that this molecular tether is a single contiguous helix that spans residues 283–300 [11, 12], although precise molecular details will have to await direct structure determination. We propose that CC/L2/SPRY packing limits the flexibility and range of orientations that the two SPRY domains can adopt relative to each other, and that this tethering mechanism significantly contributes to the avidity gains observed upon multivalent binding of TRIM5α to retroviral capsids by allowing more precise (or tailored) matching with the spacing of binding epitopes on the capsid surface. In support of this model, we identified mutations (I193A and E201A) within the putative CC/L2/SPRY packing interface that did not significantly affect dimerization or higher-order assembly but still abrogated capsid binding *in vitro* and disrupted restriction activity in cells. The simplest explanation of our data is that the impaired restriction activities of these two mutants arise from impaired CC/L2/SPRY packing. Our studies therefore support the proposed “minimum design feature” of capsid-dependent restriction factors [12, 42, 43], in which the minimal capsid-binding unit is a dimer. Higher-order assembly of the TRIM hexagonal lattice further amplifies affinity, both by spreading the interactions across the entire capsid surface and by matching the rotations of the subunits in the capsid lattice. The basal positioning mechanism occurs in context of the dimer, however, which explains the observation that TRIM5α proteins impaired in higher-order assembly (e.g., B-box mutants or deletions) can still retain the ability to bind CA tubes or other capsid mimics *in vitro*, provided that the reagents are supplied at high enough concentrations in the binding reactions [3, 10, 15, 44]. We also note that this model is compatible with the ability of a single TRIM5α protein to restrict multiple different retroviruses, because even though CA proteins have widely divergent sequences, retroviral capsids have the same underlying hexagonal arrangement with a conserved lattice spacing. Interestingly, the relative spacings of interaction modules have been found to play an important role in defining binding specificity in some avidity-driven systems [45–47]. Capsid binding specificity is dictated by the SPRY domain, and we speculate that coiled-coil/SPRY packing may contribute to specificity because productive recognition will still not occur if otherwise compatible binding epitopes on the surface of a capsid are not within the reach of allowable SPRY spacings and orientations. Testing this model will require a more precise understanding of the local SPRY/CA contacts than currently known.

It is notable that the I193A and E201A mutations did not abolish restriction activity of TRIM5α, despite apparently causing complete loss of binding in our centrifugation assay. Because the I193A and E201A mutant proteins were not impaired in higher-order assembly, our interpretation of these results is that the mutants still retained some capsid-binding activity in cells that was amplified by multivalent clustering and hexagonal lattice formation (and perhaps also further mitigated by high expression levels). Thus, SPRY/coiled-coil interactions do not appear to be fundamentally essential to recognition, but rather help to optimize avidity and maximize binding efficiency. This is consistent with studies showing that artificial restriction factors can be created by appending exogenous capsid-binding domains to the TRIM5α tripartite motif [48–50]. In these artificial systems, it is unlikely that the exogenous domains are tethered in the same manner as the native SPRY domain, yet the avidity afforded by multivalency is sufficient to generate measurable anti-viral activity.

An interesting counter-example is TRIMCyp, which contains the TRIM5α tripartite motif but harbors a cyclophilin domain for capsid binding instead of SPRY [2]. The L2 linker in TRIMCyp retains the C-terminal helical segment, but the helix is followed by an additional 11 random coil residues such that the two cyclophilin domains are likely to be flexibly connected to the coiled-coil [12]. We suggest that this high degree of flexibility is compensated for by both higher-order assembly and the significantly higher intrinsic affinity of the cyclophilin fold for the HIV-1 CA protein (with a dissociation constant of about 10 μM [36–40]). Consistent with this idea, we found that the I192A and E200A mutations had no effect on TRIMCyp’s restriction activity. The degree of affinity amplification required by TRIMCyp to allow for functional capsid recognition therefore appears to be more relaxed compared to TRIM5α.

## Materials and methods

### Protein expression and purification

Recombinant TRIM5-21R and TRIM5α_133–300_ (CC-L2) proteins were expressed and purified as described [9–11]. Briefly, TRIM5α_133–300_ proteins were expressed as a His-SUMO-tagged construct in *E*. *coli* BL21(DE3) cells using the autoinduction method [51]. The tagged construct was initially purified on Ni-NTA resin (Qiagen), the His-SUMO leader sequence was cleaved with Ulp1 protease, and the released protein was purified to homogeneity by using anion exchange and size exclusion chromatography. TRIM5-21R proteins were expressed in Sf9 cells with a Strep-FLAG leader sequence. The tagged construct was initially purified on StrepTactin resin (GE Healthcare), the leader sequence was removed by incubation with Prescission protease (GE Healthcare), and the protein purified to homogeneity using anion exchange and size exclusion. The latter two steps were particularly important in separating contaminating monomers from the dimer fraction that we used for these studies (S3A and S3B Fig). TRIM5α_133–497_ (CC-L2-SPRY) was made from the TRIM5-21R construct by deleting the RING and B-box 2 domains, expressed in Sf9 cells, and purified in the same manner as the full-length protein. All single point mutations were introduced into the appropriate constructs using the Quikchange method (Agilent) and mutants were expressed and purified in the same way as their respective wildtype constructs. Given the established propensity of TRIM5 proteins to spontaneously aggregate or assemble *in vitro*, biochemical assays were performed as soon as possible after purification. SPRY constructs lacking the V1 loop were expressed as His-GB1 fusion proteins (derived from a plasmid kindly provided by D. Ivanov) and purified as described [30].

### Electron paramagnetic resonance (EPR) experiments

Purified TRIM5α_133–300_ harboring the W196C, D288C, E292C, and W300C mutations were briefly incubated with excess dithiothreitol to reduce the exogenous cysteines, then exchanged into labeling buffer (20 mM Tris, pH 8, 100 mM NaCl) using a desalting column. The proteins were then incubated with excess MTSL (*S*-(1-oxyl-2,2,5,5-tetramethyl-2,5-dihydro-1H-pyrrol-3-yl)methylmethanesulfonothioate) overnight at 4°C. After removing unreacted label with a desalting column, the labeled proteins were concentrated to 20 μM. Deuterated glycerol (Cambridge Isotope Laboratories) at approximately 10% final concentration was added to the samples before freezing. DEER EPR measurements were performed and analyzed as described previously [52]. In brief, samples were placed inside quartz capillaries and frozen using a dry ice/isopropanol bath. Standard four-pulse DEER measurements [53] were performed with a Q-band Bruker Elexsys E580 spectrometer and EN5107D2 dielectric resonator (Bruker Biospin). Dipolar evolution data were processed and distance distributions determined using Tikhonov regularization, as implemented in the DeerAnalysis2015 software package [54]. The validation route in DeerAnalysis was used to estimate uncertainty in the distance distributions due to subtraction of the background form factor. Distance estimates from the molecular model of CC-L2 were made using the PyMol plug-in MTSSLWizard [55].

### Differential scanning fluorimetry

Thermofluor melting assays were performed as previously described [11], with final protein concentrations of 1 mg/mL for TRIM5-21R constructs and 2 mg/mL for CC-L2, CC-L2-SPRY, and SPRY constructs. Each sample was set-up in 3 or 4 replicates, and melting curves for each protein were determined at least twice, with independent protein preparations.

### NMR spectroscopy

SPRYΔV1 constructs for NMR experiments were uniformly labeled with ^15^N and/or ^13^C by growing transformed bacteria in minimal media supplemented with ^15^NH_4_Cl and/or ^13^C-glucose. Assignments for SPRY_292-497_ were kindly provided by D. Ivanov [30]. Resonance assignments (including all N-terminal residues) were independently determined for SPRY_281-497_ by using the following experiments: ^15^N/^1^H HSQC [56], CBCA(CO)NH [57], HNCA [58], HNCACB [59], HNCO [58], HNCOCA [60]. NOE cross-peaks were obtained from an ^15^N-edited NOESY-HSQC [56, 61]. Spectra were recorded on a Varian Inova 600 MHz spectrometer, processed with NMRPipe [62], and analyzed using the tools in SPARKY [63]. Chemical shift indices were calculated using the program PREDITOR [64]. Normalized chemical shift changes were calculated as described [65].

### Computational modeling

The TRIM5α coiled-coil/L2/SPRY model was built as described in the main text, using PyMol software (Schrödinger Scientific).

### SEC-MALS

These experiments were performed as described [15]. Purified CC-L2 mutants (50 μL) were injected into the column at 0.5–1 mg/mL concentrations.

### Capsid binding assays

*In vitro* pull-down assays with disulfide-stabilized HIV-1 CA tubes and purified TRIM5-21R proteins were performed as described [15].

### *In vitro* assembly of TRIM5-21R

Assembly and negative stain electron microscopy imaging of the I193A and E201A TRIM5-21R mutants were performed as described [10].

### Cell culture reagents and methods

The hemagglutinin (HA)-tagged and/or yellow fluorescent protein (YFP)-labeled rhesus TRIM5α and owl monkey TRIMCyp constructs were generated as previously described [27]. Overlapping PCR was used to generate single point mutations in the coiled-coil regions. HeLa and human embryonic kidney 293T (HEK293T) cells (from an already existing collection in-house) were cultured in complete Dulbecco’s modified Eagle’s medium (DMEM) containing 10% fetal bovine serum, penicillin (100 U/mL), and streptomycin (100 μg/mL). Vectors expressing YFP- or HA-tagged rhesus TRIM5α were made by transfecting 293T cells with the respective wildtype and mutant TRIM5 plasmids along with VSV-G and pCig-B. HeLa cells stably expressing the YFP-tagged proteins were generated by G418 (400 μg/mL) selection at 48 h post-transduction. The stable cell lines were then analyzed by immunofluorescence and western blotting.

### Cytoplasmic body assembly assays

HeLa cells (from an already existing collection in-house) stably expressing YFP-tagged TRIM5α proteins were plated onto fibronectin-treated coverslips, allowed to adhere, and fixed with 3.7% formaldehyde and stained with DAPI. Images were collected with a DeltaVision microscope (Applied Precision) equipped with a digital camera (CoolSNAP HQ; Photometrics), using a 1.4 numerical aperture objective lens, and were deconvolved with SoftWoRx deconvolution software (Applied Precision). *Z*-stack images of each cell line were acquired by using identical acquisition parameters. Deconvolved images were analyzed for fluorescent cytoplasmic bodies by using the Surface Finder function of the Imaris software package (Bitplane).

### Restriction assays

Vesicular stomatitis virus G protein (VSV-G)-pseudotyped R7ΔEnv HIV-GFP was produced by transfecting HEK293T cells as previously described [66]. Virus infectivity was assessed by infecting equivalent numbers of cells in a 24-well plate, and green fluorescent protein (GFP) expression was determined at 48h post-infection by using a FACSCanto II flow cytometer (Becton, Dickinson).

## Supporting information

S1 Fig Raw DEER data. Left panels: Uncorrected DEER traces, V(t)/V(0), for each of the four mutants. The mutant W196R1 that is labeled within the coiled-coil helices produce a noticeable DEER echo, indicating a well-defined distance. Red lines indicate the background form factor. Right panels: Corrected dipolar evolution curves, F(t)/F(0), after subtraction of the background form factor. The red traces represent the best fits to the DEER data, and yield the distributions shown in Fig 1.
(TIFF)

S2 Fig Overlayed ^15^N-^1^H HSQC spectra showing backbone amide footprints of indicated SPRY domain constructs. Top panel: Residues that undergo the largest chemical shift changes (apart from N-terminal residues) in comparing the two constructs are encircled and labeled. Bottom panel: Enlarged view of central region containing helical resonances, with complete residue assignments for the longer construct (SPRY_281-497_).
(TIFF)

S3 Fig Oligomerization status of TRIM5-21R proteins during purification. (A) Anion exchange chromatography reveals the relative fractions of monomer and dimer species at the start of purification. Wildtype TRIM5-21R elutes as a doublet peak, with the minor monomer fraction eluting early and the major dimer fraction eluting late. Blue curve = UV absorbance trace. Brown curve = conductivity trace arising from application of a linear salt gradient. (B) Size exclusion chromatography of pooled anion exchange fractions allows further separation of contaminating monomers from the desired dimer species. (C) Representative anion exchange profiles of class I mutants indicate significantly elevated monomer fractions. (D) Representative anion exchange profiles of class II mutants.
(TIFF)

S4 Fig Cytoplasmic bodies formed by wildtype control rhesus YFP-TRIM5α and class I mutants. (A-D) Representative images of HeLa cells stably expressing the indicated constructs. Cytoplasmic bodies appear as green puncta. DAPI was used to stain nuclei blue. Scale bars = 10 μ. (E) The number of cytoplasmic bodies was counted in each cell and normalized to the intracellular YFP concentration.
(TIFF)
