# Supplementary figures and images for: TRIM5α SPRY/coiled-coil interactions optimize avid retroviral capsid recognition
Source: PLoS Pathog. 2017 Oct 17;13(10):e1006686. doi: 10.1371/journal.ppat.1006686 (PMC5667893; doi:10.1371/journal.ppat.1006686)

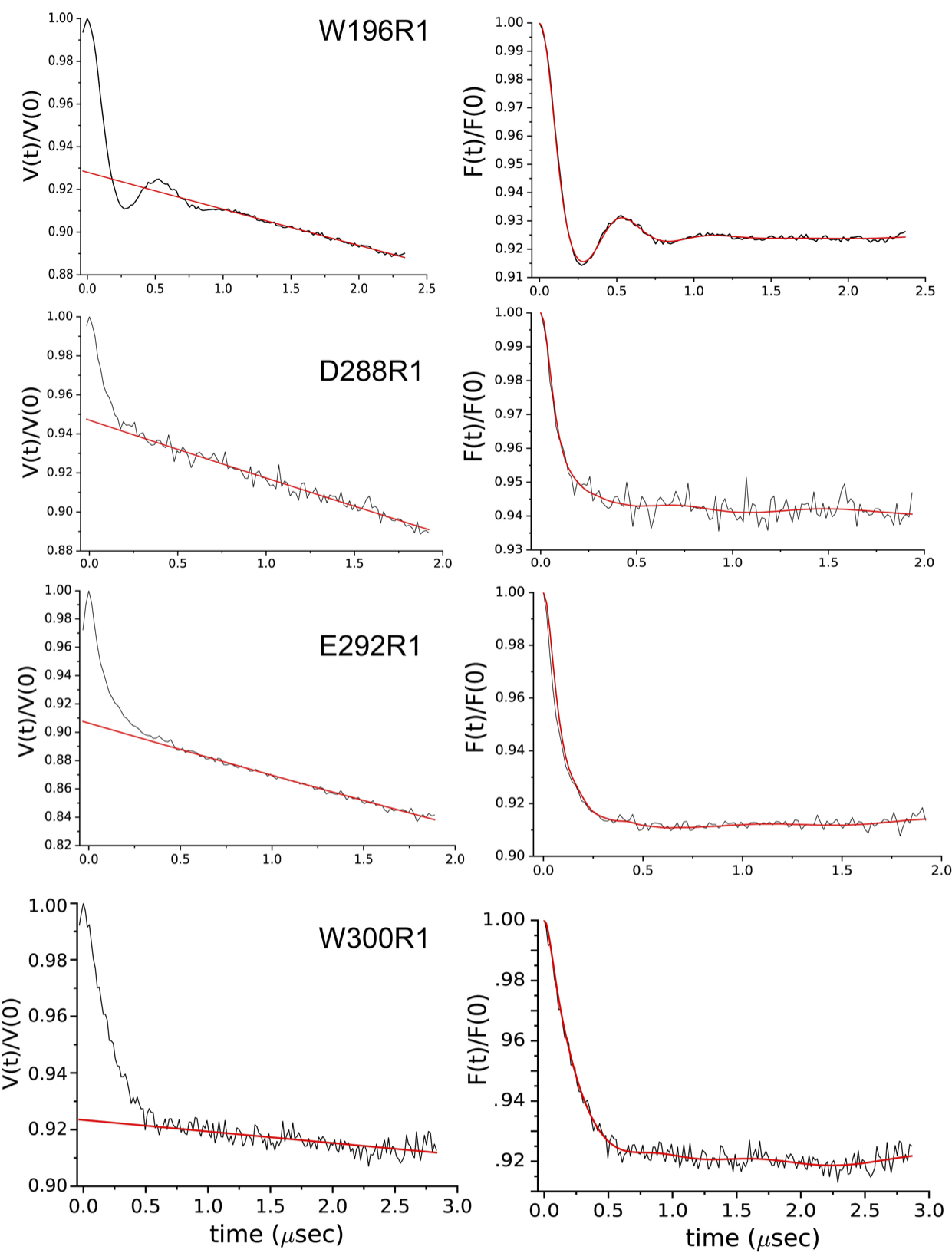

Supplement: S1 Fig — Left panels: Uncorrected DEER traces, V(t)/V(0), for each of the four mutants. The mutant W196R1 that is labeled within the coiled-coil helices produce a noticeable DEER echo, indicating a well-defined distance. Red lines indicate the background form factor. Right panels: Corrected dipolar evolution curves, F(t)/F(0), after subtraction of the background form factor. The red traces represent the best fits to the DEER data, and yield the distributions shown in Fig 1. (TIFF) [file ppat.1006686.s001.tiff]

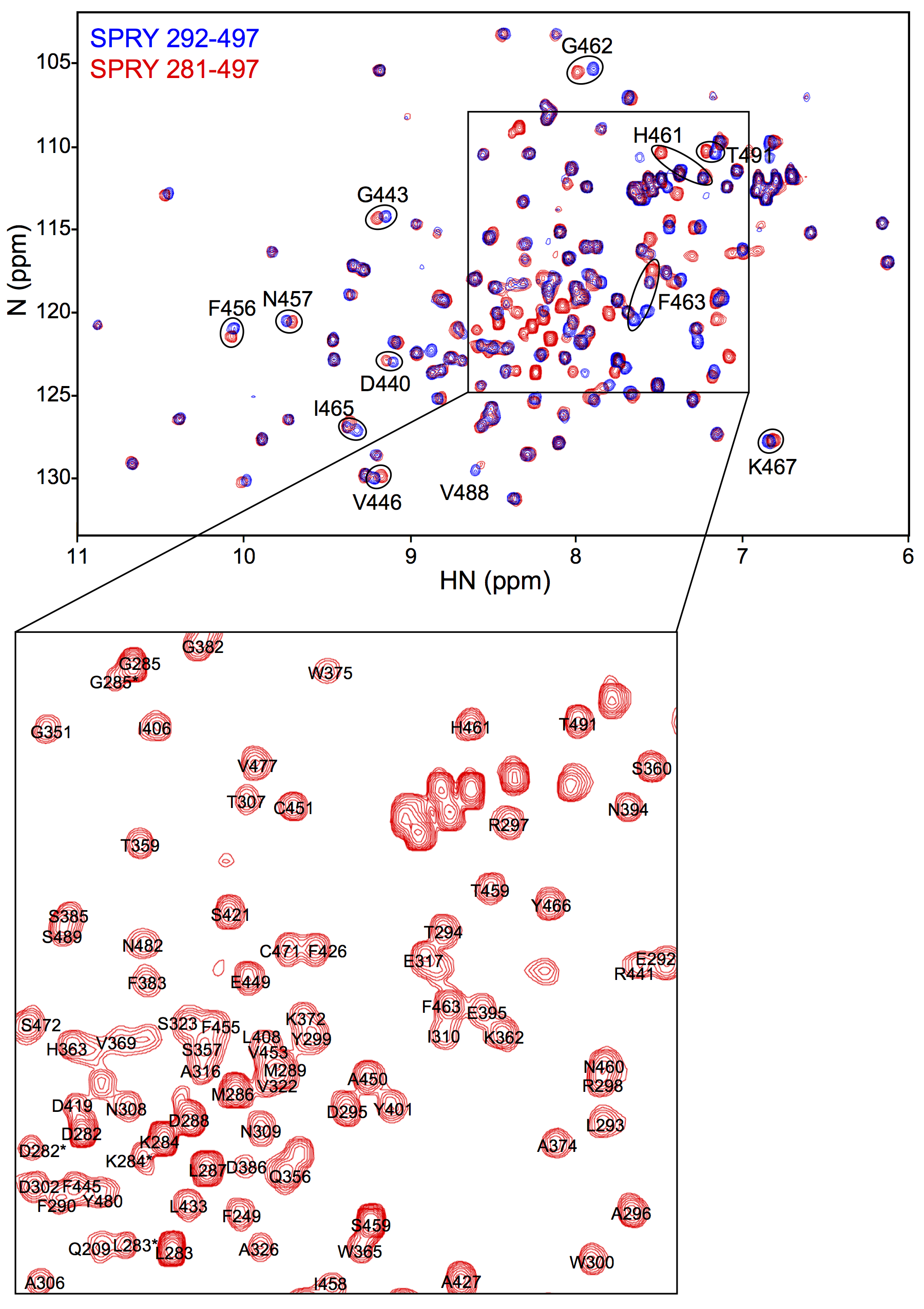

Supplement: S2 Fig — Top panel: Residues that undergo the largest chemical shift changes (apart from N-terminal residues) in comparing the two constructs are encircled and labeled. Bottom panel: Enlarged view of central region containing helical resonances, with complete residue assignments for the longer construct (SPRY281-497). (TIFF) [file ppat.1006686.s002.tiff]

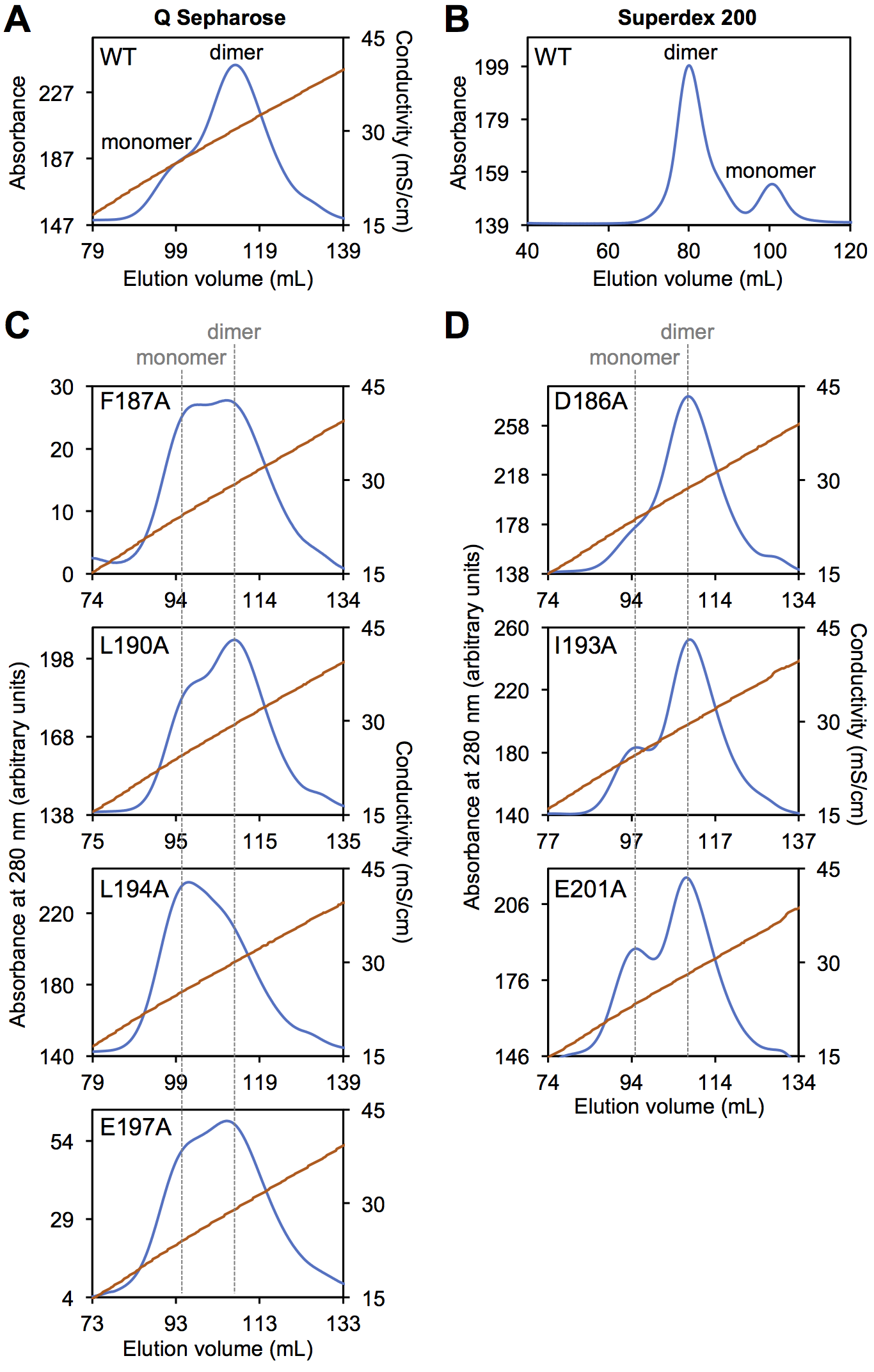

Supplement: S3 Fig — (A) Anion exchange chromatography reveals the relative fractions of monomer and dimer species at the start of purification. Wildtype TRIM5-21R elutes as a doublet peak, with the minor monomer fraction eluting early and the major dimer fraction eluting late. Blue curve = UV absorbance trace. Brown curve = conductivity trace arising from application of a linear salt gradient. (B) Size exclusion chromatography of pooled anion exchange fractions allows further separation of contaminating monomers from the desired dimer species. (C) Representative anion exchange profiles of class I mutants indicate significantly elevated monomer fractions. (D) Representative anion exchange profiles of class II mutants. (TIFF) [file ppat.1006686.s003.tiff]

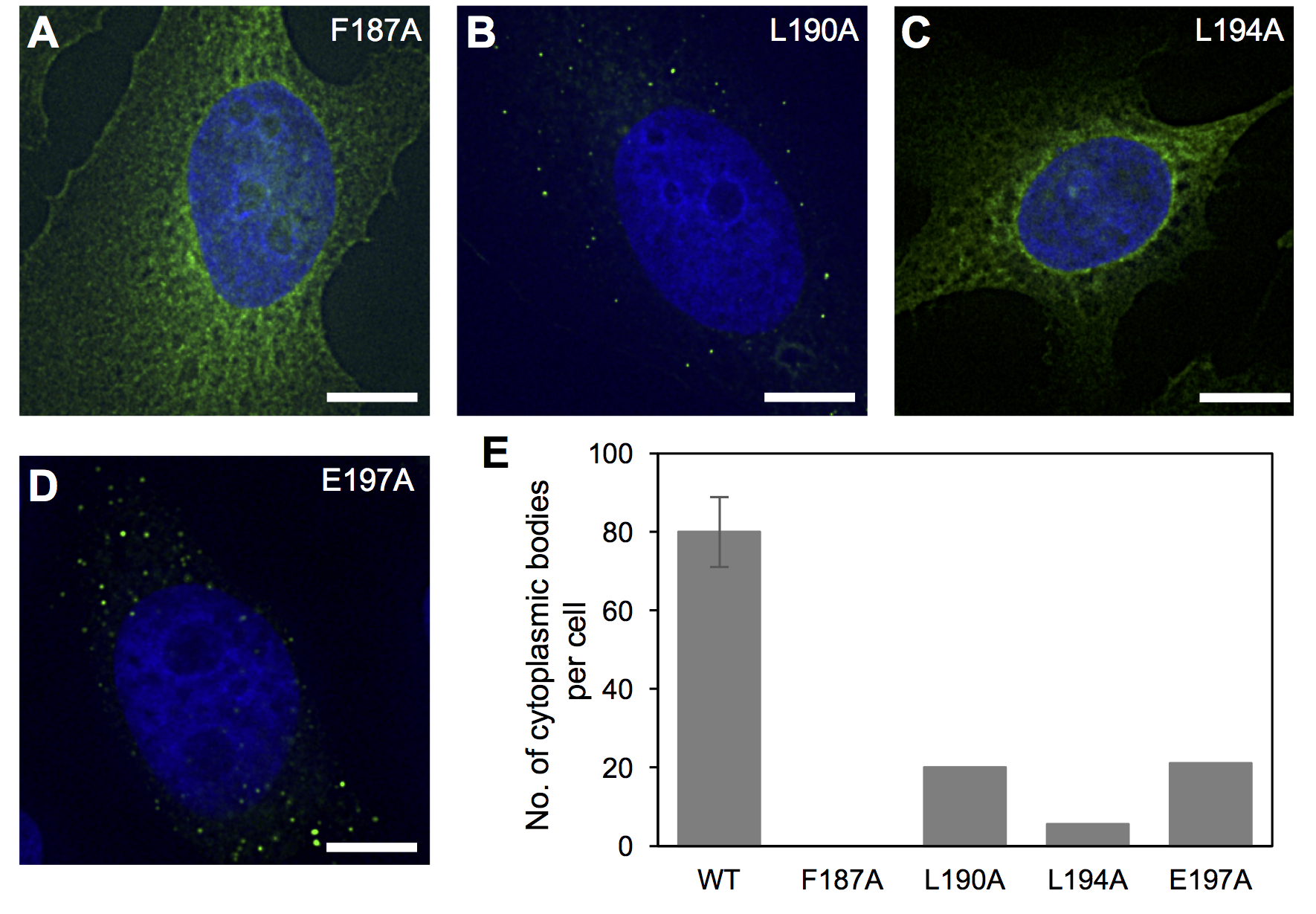

Supplement: S4 Fig — (A-D) Representative images of HeLa cells stably expressing the indicated constructs. Cytoplasmic bodies appear as green puncta. DAPI was used to stain nuclei blue. Scale bars = 10 μ. (E) The number of cytoplasmic bodies was counted in each cell and normalized to the intracellular YFP concentration. (TIFF) [file ppat.1006686.s004.tiff]
